# Interactions with and activation of immune cells by CD41a^+^ extracellular vesicles

**DOI:** 10.3389/fimmu.2025.1509078

**Published:** 2025-02-14

**Authors:** Marie Tamagne, Mehdi Khelfa, Souganya Many, Deborah Neyrinck-Leglantier, Adèle Silane Delorme, Marion Klea Pinheiro, Muriel Andrieu, Sabine Cleophax, France Pirenne, Benoît Vingert

**Affiliations:** ^1^ Univ Paris Est Creteil, INSERM, IMRB, Creteil, France; ^2^ Etablissement Français du Sang, Ivry sur Seine, France; ^3^ Institut Cochin, Inserm U1016, Centre National de la Recherche Scientifique (CNRS) UMR8104, Université Paris-Cité, Paris, France

**Keywords:** extracellular vesicles (EVs), immune activation, platelet transfusion, immunomodulation, multi-omic analyses

## Abstract

**Introduction:**

The immunological profiles of CD4^+^ T lymphocytes (TLs) from patients with hematological malignancies differ between patients who have and have not received transfusions. There may be several reasons for these differences, including the presence of extracellular vesicles (EVs) derived from plasma membrane budding and present in the platelet concentrates. Indeed, EVs can modulate the immune system through interactions with many immune cells, but the underlying mechanisms remain incompletely understood.

**Methods:**

We therefore investigated how interactions with CD41a^+^ EVs cause immune cells to change phenotype and function. CD41a^+^ EVs were cultured with TLs, B lymphocytes, and monocytes. Given the potential involvement of monocytes in leukemia progression, we performed a new original multi-omics study to confirm the protein changes and gene activation observed following interaction with CD41a^+^ EVs

**Results:**

The CD41a^+^ EVs had immunomodulatory effects on all these cell types but this effect depended on the numbers of EVs. CD4^+^ TLs required large numbers of CD41a^+^ EVs for activation, whereas monocytes were the most sensitive. With the new multi-omics technique, we confirmed the direct effects of CD41a^+^ EVs on protein phenotype and gene activation.

**Conclusion:**

Transfusion EVs should be considered during the immunological follow-up of patients after transfusion to detect immunological effects on malignant hemopathies, and during the development of new immunotherapies.

## Introduction

Transfusions of red blood cells, platelets or plasma exchange are not always immunologically innocuous to patients. They have important immunomodulatory effects. In practice, alloimmunization is the first element studied, as it is characterized by the appearance of alloantibodies, particularly in the context of polytransfusion. Alloantibodies can affect patient survival (1, 2), particularly in patients on chemotherapy (3).

Fortunately, alloimmunization is not systematic (4, 5) and the presence of alloantibodies is not necessarily associated with a state of refractoriness (6).

Immunological consequences of transfusion independent of alloimmunization have long been described in a number of diseases, including transfusion-related immunomodulation syndrome (TRIM) and transfusion-related acute lung injury (TRALI) (7, 8). Questions have been raised about the clinical importance of these consequences, particularly for TRIM syndrome in cancers (9).

Much more is known about alloimmunization. CD4^+^ T lymphocytes (TLs) are known to play a key role (10–12), but little is known about the role in human alloimmunization of antigen-presenting cells (dendritic cells or monocytes) and effector cells (i.e., B lymphocytes (BLs)) (13–15). Other immune cells, such as macrophages, neutrophils and NKs cells may also underlie immunomodulation following transfusion (7, 8, 16).

The paucity of information concerning the immunomodulatory effects of transfusions in humans may seem surprising, particularly given the importance of understanding these effects for the correct immunological management of patients, but new information is emerging, particularly in malignancies diseases (12).

One of the major immunoregulatory elements present in blood products is the extracellular vesicles (EVs) derived from cell membrane budding (17–30).

EVs can modulate the immune system through interactions with many immune cells: conventional CD4^+^ TLs, follicular helper CD4^+^ TLs (Tfh), IL17^+^CD4^+^ TLs (Th17), regulatory CD4^+^ TLs (Treg), monocytes, BLs, and dendritic cells (DC) (17, 21–28). These EVs are between 160 and 900 nm in diameter and are also known as microparticles (MPs) or ectosomes, to differentiate them from exosomes. Their size makes it possible to phenotype or purify them by flow cytometry. Exosomes are smaller (40-100 nm in diameter) and are derived from intracellular membrane compartments. It is not yet possible to study them reliably by flow cytometry. In this study, we therefore focused exclusively on the largest vesicles, referred to as EVs in this manuscript, and did not consider exosomes.

Platelet-derived EVs form the largest subset of EVs in platelet concentrate (PC), but not all of the EVs present in PCs are derived from platelets (29). These platelet-derived EVs, more commonly referred to as platelet-derived microparticles (PMPs), are often isolated from platelet concentrates and are not generated exclusively by budding of the platelet membrane. Care is therefore required when reading publications on this subject. These PMPs express many membrane proteins and carry some of the cytoplasm from their cells of origin (17, 30). They may therefore contain RNA, soluble factors, cytokines, and organelles and play an important role in immunomodulatory processes (17, 24–26, 28–30).

The interactions of EVs with immune cells have been little described since the first studies of chemokine receptor transfer in HIV (31, 32). Nevertheless, several major studies have been published on EV interactions with endothelial cells (33, 34), and, more particularly, on the interactions of PMPs with Tregs (24), neutrophils (35), and macrophages (36, 37). These interactions and their reprogramming are already the subject of research with the aim of developing new therapies based on monocyte reprogramming for cancer immunotherapy (38).

In this study, we investigated the interaction of CD41a^+^ EVs with CD4^+^ TLs, BLs, and monocytes, and assessed the changes in phenotype and function of these cells. EVs from transfusions are known to be involved in intercellular communication, modulating the immune system (17, 21–28). The underlying mechanisms remain incompletely understood, but these immune cell interactions may involve immune ligands/receptors present on the surface of the EVs. We recently showed that CD27^+^ and CD70^+^ EVs can transfer these receptors to CD4^+^ TLs, thereby increasing activation and lymphoproliferation (39). Platelets do not express CD27 or CD70, but they do express many other molecules, including Toll-like receptors, major histocompatibility complex class I, CD40, CD40 ligand (CD40L, CD154), OX40 (CD134) and OX40 ligand (OX40L, CD252) (40–43).

We chose to study these CD41a^+^ EVs in plasma because these vesicles have a high prevalence in plasma and, blood products, and because platelets are immune system cells that can express these molecules at sufficiently high levels for interaction with other immune cells (40–43).

CD41a^+^ EVs were purified by flow cytometry sorting and cultured, at various ratios, with cell preparations enriched in CD4^+^ TLs, BLs, and monocytes by magnetic cell purification. The ratios of cells to EVs studied here were based on the numbers of CD41^+^ EVs in platelet concentrates and of polytransfusions in patients with hematological malignancies (12, 29). Functional studies were performed after incubating the cells with the EVs. Interactions (particularly with antigen-presenting cells) were observed, resulting in phenotypic and functional changes to all the cell types studied, including monocytes, which are thought to be involved in leukemia progression (44, 45). Significant interactions between EVs and monocytes have been reported in previous studies (25, 46). We therefore developed a new method for analyzing the interactions of EVs with cells based on original multi-omics approaches with EV-labeled oligonucleotide-conjugated antibodies and purified monocytes. The results obtained with this new method confirm that cells are strongly activated by EVs — in this case by CD41a^+^ EVs — leading to significant changes in phenotype, protein content and gene activation in the cells (here, monocytes). However, the effects of these interactions are strongest for antigen-presenting cells, opening up new possibilities for treatment approaches (38) and highlighting the importance of vigilance regarding the immunological effect of transfusion in patients with malignant hemopathies (12).

## Materials and methods

### Biological samples

Blood samples were collected from healthy donors (HDs). For the isolation of PBMCs, blood samples were collected in tubes containing sodium heparin (BD Biosciences, Franklin Lakes, NJ). For EV phenotyping and the isolation of CD41a-expressing EVs, blood samples were collected in tubes containing acid citrate dextrose solution B (ACD-B) (BD Biosciences). All blood samples were provided by the French national blood bank (*Etablissement Français du Sang*, EFS).

None of the HDs had suffered an infection (bacterial, viral, fungal, yeast) or been vaccinated in the 30 days preceding inclusion, and none had received a platelet transfusion. All the participants gave written informed consent.

### EV-enriched preparation

EV-enriched preparations were obtained by differential centrifugation. As previously described, blood samples were centrifuged at an initial speed of 3,000 x *g* (10 minutes) (22, 23, 29, 39, 47). The plasma thus obtained was centrifuged at 13,000 x *g* (10 minutes) for the preparation of a platelet-free supernatant. EVs were concentrated by centrifuging the platelet-free supernatant for 1 hour at 100,000 x *g* (4°C). They were then resuspended in filter-sterilized (passage through a filter with 0.1 μm pores) PBS for flow cytometry.

### EV phenotyping

EVs were labeled with fluorochrome-conjugated monoclonal antibodies. Fluorescence was assessed with a 20-parameter LSR Fortessa flow cytometer with a small-particle option (BD Biosciences, San Jose, CA) based on photomultiplier (PMT)-coupled forward scatter (FSC) detection. This mode of detection was used to ensure the optimal detection of EVs with diameters of 200 to 900 nm. The performance of the flow cytometer was checked before each assay. EV flow cytometry assays were performed in accordance with the guidelines of the International Society for Extracellular Vesicles (48, 49). Megamix-Plus FSC and SSC beads (BioCytex, Marseille, France) of known dimensions (beads with diameters ranging from 200 nm to 900 nm) were used to standardize the FSC-PMT parameters and define the EV gate. EVs were labeled with anti-CD41a APC-H7, anti-CD62P BUV395, anti-CD73 BB515, anti-CD86 AF700, anti-OX40L PE, anti-OX40 FITC, anti-CD40L PE-CF594, anti CD80 BV605, anti-CD40 AF700 (BD Biosciences), anti-CD107 BV785 and anti-CLEC2 PE (Biolegend, San Diego, CA) antibodies.

### EV sorting by flow cytometry

In this study, for all functional assays, EVs were sorted as previously described with a MoFlo Astrios cell sorter (Beckman Coulter, Brea, CA) equipped with a PMT-FSC detector (23, 39). Flow cytometer performance was assessed before the cell-sorting experiments. Polystyrene beads (FSC plus Megamix, BioCytex) of known dimensions (200 nm, 500 nm, and 900 nm in diameter) were used to standardize PMT-FSC parameters and to define the total EV gate. The sensitivity of vesicle detection was also checked with silica beads (ApogeeFlow beads, Hertfordshire, United Kingdom). EVs were acquired and purified at low speed (200 evt/s).

For CD41a^+^ EV sorting by flow cytometry, EVs were labeled with anti-CD41a APC-H7 antibody (BD Biosciences). We used a commercial kit to check for the absence of endotoxin in purified EV preparations (Invivogen, San Diego, CA).

### Assay of CD41a^+^ EV binding to immune cells

#### PBMCs were isolated by Ficoll density gradient centrifugation

CD4^+^ TLs, CD8^+^ TLs, monocytes, and B cells were purified from PBMCs by magnetic isolation with anti-human CD4 (#557939), anti-human CD8 (#557941), anti-human CD14 (#558454 plus #51-9004594), and anti-human CD19 antibody-conjugated magnetic particles (551520), respectively (BD Imag, BD Biosciences).

We cultured 5 x 10^5^ cells for 18 hours with quantified sorted CD41a-expressing EVs. The EVs were added to the culture at ratios of 1:2, 1:10, 1:20 or 1:100 (cells: EVs) in filter-sterilized (passage through a filter with 0.1 µm pores) culture medium. As a control, cells were also cultured without EVs. The culture medium consisted of RPMI 1640 supplemented with 5% FBS (Dutscher, Bernolsheim, France), 2 mM L-glutamine, 100 µg/ml penicillin/streptomycin, MEM non-essential amino acids solution (1X), and 1 mM sodium pyruvate (all from Thermo Fisher Scientific, Waltham, MA).

After coculture, cells were harvested and stained with anti-CD4 BUV395, anti-CD8 BUV737, anti-CD19 PE-Cy7, (BD Biosciences) or anti-CD14 PE (Biolegend) antibodies for the assessment of CD41a co-expression (labeling with an anti-CD41a APC-H7 antibody during EV sorting) by flow cytometry.

### Cell activation

We investigated interactions with CD41a-expressing EVs and the resulting cell activation in CD4^+^ TLs, monocytes, and BLs cultured for 18 h in the presence or absence of purified CD41a^+^ EVs at a ratio of 1:20 (cells: EVs). The cells that interacted with CD41a-expressing EVs were collected by flow cytometry, with a minimum of 500 CD41a^+^ cells collected for each cell subpopulation.

CD4^+^ TLs and monocytes were stained with the antibodies described in **Supplementary Table 1** and were then fixed and permeabilized with the Fix & Perm kit (Thermo Fisher Scientific) according to the manufacturer’s instructions. Intracellular markers were detected with the antibodies described in **Supplementary Table 1**. Aqua Live/Dead viability dye (Thermo Fisher Scientific) was added to exclude dead cells.

BLs were stained with the antibodies described in **Supplementary Table 1** for the investigation of membrane markers and to define B lymphocyte subpopulations: naïve B cells (IgD^+^CD27^-^), transitional B cells (IgD^+^CD27^-^CD24^hi^CD38^hi^), marginal zone B cells (IgD^+^CD27^+^), memory B cells (IgD^-^CD27^+^CD24^+^CD38^lo^), plasmablasts (IgD^-^CD27^+^CD24^lo^CD38^hig^) and IgD^-^CD27^-^ B cells. The supernatant was removed and frozen at -20°C for assessments of immunoglobulin secretion. Multiplex assays were performed with a commercial kit, according to the manufacturer’s instructions (ProcartaPlex Human Isotyping 7plex Kit, Thermo Fisher Scientific). Bead fluorescence was read with a MAGPIX reader (Luminex, Austin, TX).

### Single-cell multi-omics assay

EV enrichment was performed on HD platelet concentrates sampled at the platelet preparation laboratory of the EFS. These concentrates were centrifuged at 100,000 x *g* for 1 h at 4°C. The resulting EV-enriched preparation was resuspended in filter-sterilized (passage through a filter with 0.1 μm pores) PBS and labeled by incubation with AbSeq anti-CD41a antibody (clone HIP8, BD Biosciences) for 1 h at 4°C. The EVs were washed by centrifugation for 1 hour at 100,000 x *g* and 4°C, to eliminate the antibodies that had not bound to EVs. An aliquot of the EV-enriched preparation was stained with an AF647-coupled oligonucleotide to detect EVs stained with AbSeq antibodies for quantification in Trucount tubes (BD Biosciences).

PBMCs were isolated from buffy coats provided by EFS. Monocytes were purified by magnetic isolation from PBMCs with the Classical Monocyte Isolation Kit (Miltenyi Biotec, Bergisch Gladbach, Germany) and stained with an anti CD14-PE antibody (Biolegend) for sorting on a BD FACSAria Fusion flow cytometer (BD Biosciences).

Monocytes were cultured with EVs for 18 hours at a ratio of 1:10 (cells: EVs). The cells were then harvested and stained with 20 BD AbSeq antibodies (**Supplementary Table 2**). Targeted scRNA-seq analysis was performed, as previously described (12), with the BD Rhapsody Single-Cell Analysis System (BD Biosciences), according to the manufacturer’s instructions. The BD Human Single-Cell Multiplexing Kit was used to multiplex up to five sets of culture conditions per Rhapsody cartridge. For library construction, the samples were pooled before cartridge loading. The BD Rhapsody Immune Response Targeted Panel for Humans was used to assess mRNA levels for 399 genes (#633750).

### Single-cell multi-omics analysis

For single-cell RNAseq, analyses were performed with SeqGeq software (v1.8, BD Biosciences), and Kruskal-Wallis tests were performed to identify the differentially expressed genes and proteins. Only genes and proteins with a *Q*<0.05 were considered to be differentially expressed.

The ViolinBox plug-in (version 5.1.11, https://www.flowjo.com/exchange/#/plugin/profile?id=13) was used to generate heatmaps. ViolinBox,developed by Luthy J., Taylor I., Spidlen J, is a plug-in based on Poggiali D’s algorithm.

For each gene and protein, the mean level of expression in each of the three conditions (monocytes without EVs, monocytes that had interacted with CD41a^+^ EVs, and monocytes cultured with EVs but without CD41a^+^ EV interaction) was calculated with the ViolinBox plug-in. Expression levels cannot be compared between genes or proteins in this representation.

### Flow cytometers and fluorescence analysis

For all flow cytometry analyses, fluorescence was measured on an LSRFortessa flow cytometer (BD Biosciences). Flow cytometry data were analyzed with FlowJo software (v.10.8.1, BD Biosciences).

### Statistical analysis

All analyses were performed with Prism 6.07 software (GraphPad Software, La Jolla, CA). All significant differences between groups (*P*<0.05) are indicated on the data plots. Details of the statistical tests performed are provided in the legend to each figure.

## Results

### Interaction of CD41a^+^ EVs with immune cells

**Figure 1:**
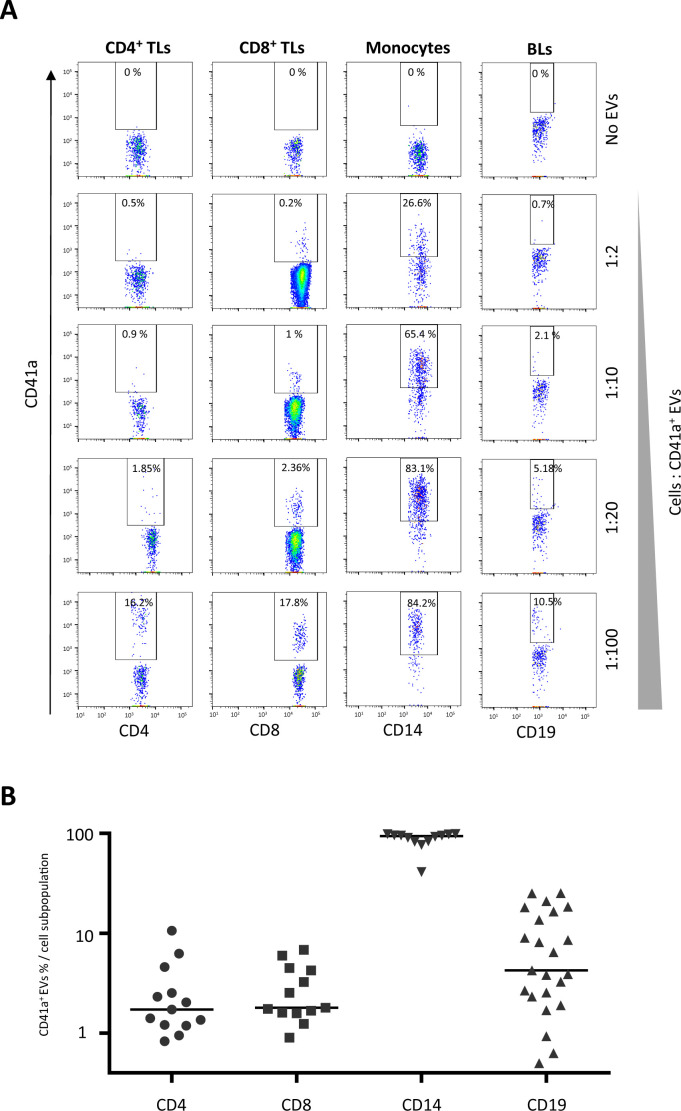
Interaction of platelet microparticles with immune cells. **(A)** Example of the gating strategy used to assess the interaction of CD41a^+^ EVs with CD4^+^ T lymphocytes (TLs), CD8^+^ TLs, monocytes (CD14^+^), or B lymphocytes (BLs, CD19^+^). Cells were cocultured for 18 hours with known numbers of CD41a^+^ EVs, at ratios of 1:2, 1:10, 1:20 and 1:100 (cells: CD41a^+^ EVs). Before culture, EVs were stained with an APC-H7-labeled anti-CD41a antibody and sorted by cytometry. Samples from 33 healthy donors (HDs) were used in 12 coculture experiments. **(B)** Percentage of cells expressing CD41a from EVs (indicating an interaction) at a coculture ratio of 1:20 (cells: CD41a^+^ EVs). Horizontal bars indicate the median value.

PBMCs were cultured with purified EVs, as previously described (12). We cultured immune cells with purified EVs to investigate the interactions of CD41a^+^ EVs with the following cells: CD4^+^ TLs, CD8^+^ TLs, monocytes (CD14^+^ cells), and BLs (CD19^+^ cells). These interactions were assessed by labeling the CD41a^+^ EVs with an anti-CD41a APC-H7 antibody (**Figure 1A**). The variation of CD41a expression on the EVs in platelet concentrates was mimicked by coculturing immune cells with different ratios of EVs to cells (**Figure 1A**).

Rates of interaction with CD41a^+^ EVs were lowest for TLs. At a ratio of 1:20 (cells: EVs), associations were observed for 2.8 ± 2.8% CD4^+^ TLs and 2.9 ± 1.9% CD8^+^ TLs (**Figure 1B**). A mean of 8.6 ± 8.2% BLs (CD19^+^ cells) interacted with CD41a^+^ EVs and the highest rates of interaction with these EVs were obtained for monocytes (CD14^+^ cells, mean of 87.3 ± 16.2%).

### Interaction of CD41a^+^ EVs with CD4^+^ TLs

**Figure 2:**
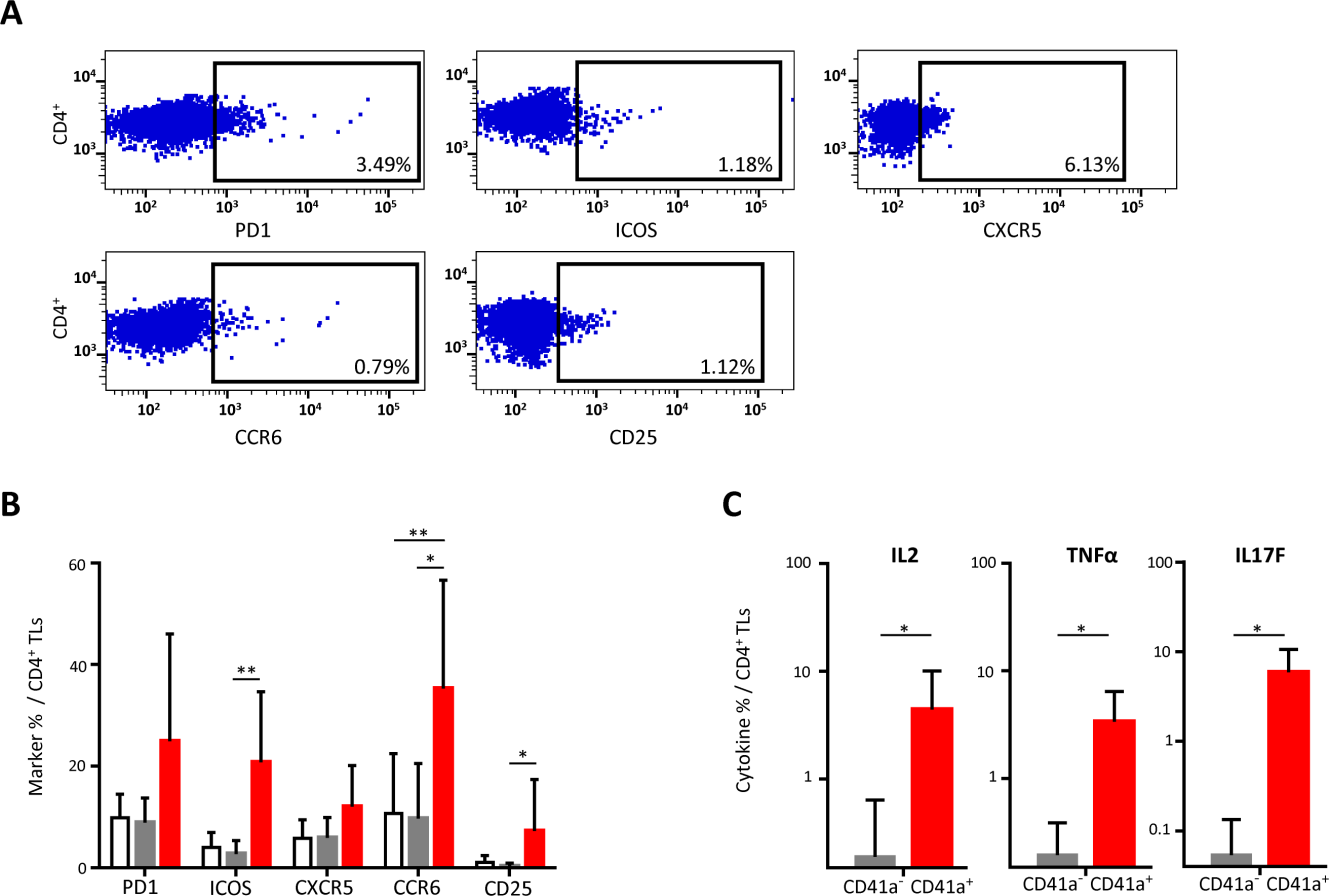
Impact of the binding of CD41a^+^ EVs to CD4^+^ TLs. CD4^+^ TLs were cocultured for 18 hours with known numbers of CD41a^+^ EVs at a ratio of 1:20 (CD4^+^ TLs: CD41a^+^ EVs). Cocultures with cells from seven HDs were assessed in five experiments. **(A)** Example of the gating strategy. **(B)** The percentage (mean ± SD) of CD4^+^CD45RA^-^PD1^+,^ CD4^+^CD45RA^-^ICOS^+^, CD4^+^CD45RA^-^CXCR5^+^, CD4^+^CD45RA^-^CCR6^+^ or CD4^+^CD45RA^-^CD25^+^ TLs was determined in the absence (white bars) or presence of EVs (CD4^+^CD41a^+^, red bars and CD4^+^CD41a^-^, gray bars). The *P* values reported were obtained in ANOVA and Friedman’s *post hoc* tests: *P<0.05, **P<0.01. **(C)** The percentages of cells secreting cytokines (mean ± SD) were determined by the intracellular staining of CD4^+^ TLs cocultured for 18 h with EVs (CD4^+^CD45RA^-^CD41a^+^, red bars and CD4^+^CD45RA^-^CD41a^-^, gray bars). The *P* values reported were obtained in Wilcoxon’s test. *P<0.05.

CD4^+^ TLs are known to be involved in alloimmunization responses. We therefore investigated the expression of cellular markers on these cells after interaction with CD41a^+^ EVs. Cells that did not interact with EVs had levels of activation marker expression similar to those of control cells cultured without EVs. By contrast, cells interacting with CD41a^+^ EVs displayed increases in the expression of PD1 and CXCR5, with significant differences for ICOS, CCR6 and CD25 (20.79 ± 13.85%, 35.28 ± 21.34% and 7.20 ± 10.16%, respectively, *P*<0.01 and *P*<0.05) (**Figures 2A, B**). This increase in the expression of activation markers on the membrane was accompanied by increases in the production of cytokines such as IL-2, TNFα and IL-17F in CD4^+^ TL cells interacting with CD41a^+^ EVs, with 4.4 ± 5.6%, 3.38 ± 3.03 and 5.88 ± 4.68% of these cells, respectively, secreting these cytokines (*P*<0.05) (**Figure 2C**).

### Interaction of CD41a^+^ EVs with BLs

**Figure 3:**
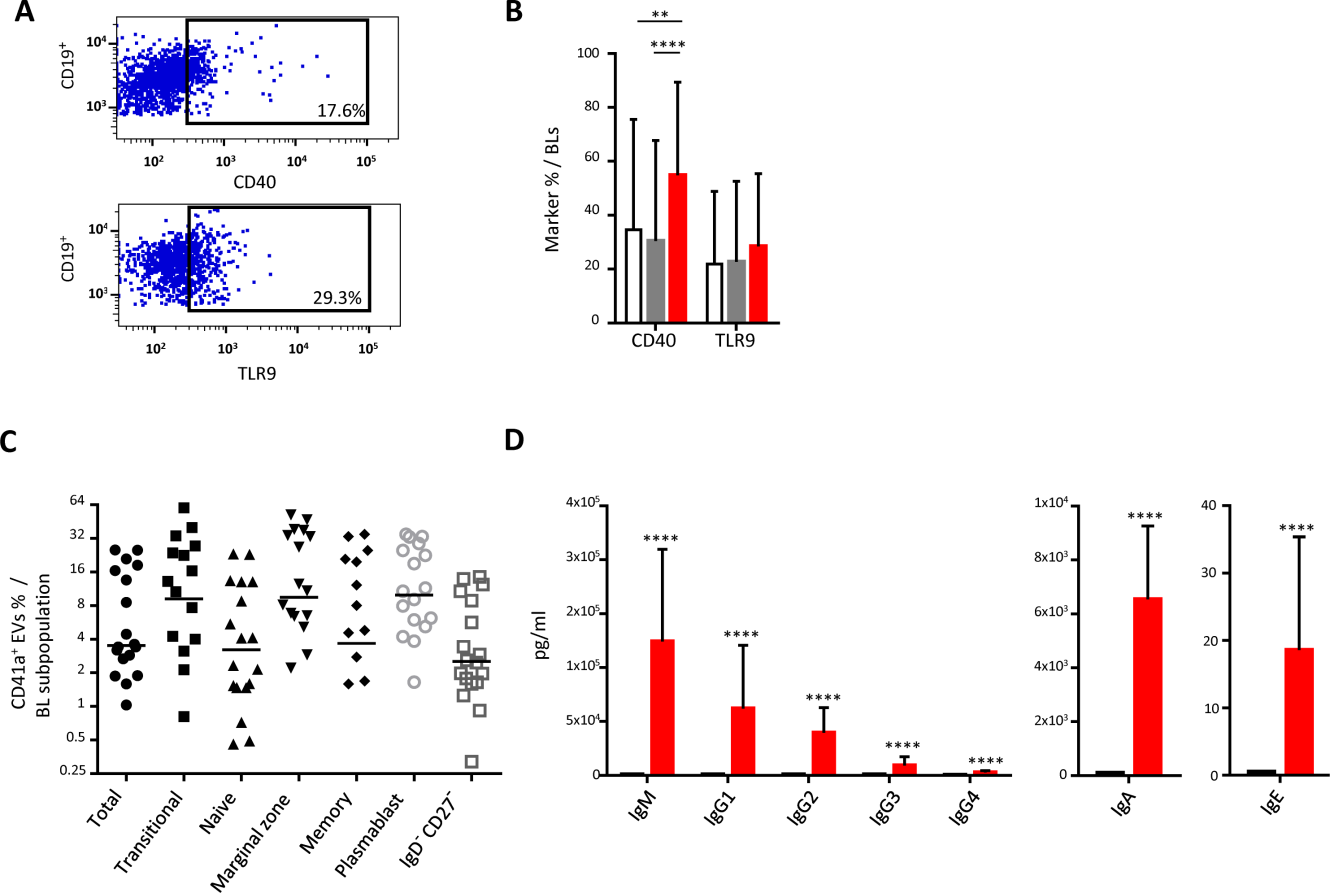
Impact of the binding of CD41a^+^ EVs to B lymphocytes. B lymphocytes (BLs) were cocultured for 18 hours with known numbers of CD41a^+^ EVs at a ratio of 1:20 (CD19^+^ cells: CD41a^+^ EVs). We used cells from 18 HDs in 10 experiments. **(A)** Example of the gating strategy. **(B)** The percentages (mean ± SD) of CD19^+^ cells expressing CD40 or TLR9 were determined in the absence (white bars) or presence of EVs (CD11c^+^CD41a^+^, red bars and CD11c^+^CD41a^-^, gray bars). The *P* values shown were obtained in ANOVA and Friedman’s *post hoc* tests: * *P*<0.05, ** *P*<0.01. **(C)** The percentage of cells expressing CD41a after coculture was determined by flow cytometry on CD19^+^ cells (●), transitional B cells (◼, IgD^+^CD27^-^CD24^hi^CD38^hi^), naïve B cells (▴, IgD^+^CD27^-^), marginal zone B cells (▾, IgD^+^CD27^+^), memory B cells (♦, IgD^-^CD27^+^CD24^+^CD38^lo^), plasmablasts (○, IgD^-^CD27^+^CD24^lo^CD38^hig^) and IgD^-^CD27^-^ B cells (□). **(D)** The secretion of total IgM/IgG (left panel), IgA (middle panel) and IgE (right panel) was assessed by Luminex methods on the culture supernatant of 2 x 10^5^ cells cultured with (red bars) or without (black bars) CD41a^+^ EVs. The *P* values shown were obtained in Wilcoxon’s test. ****P<0.0001.

**Figure 4:**
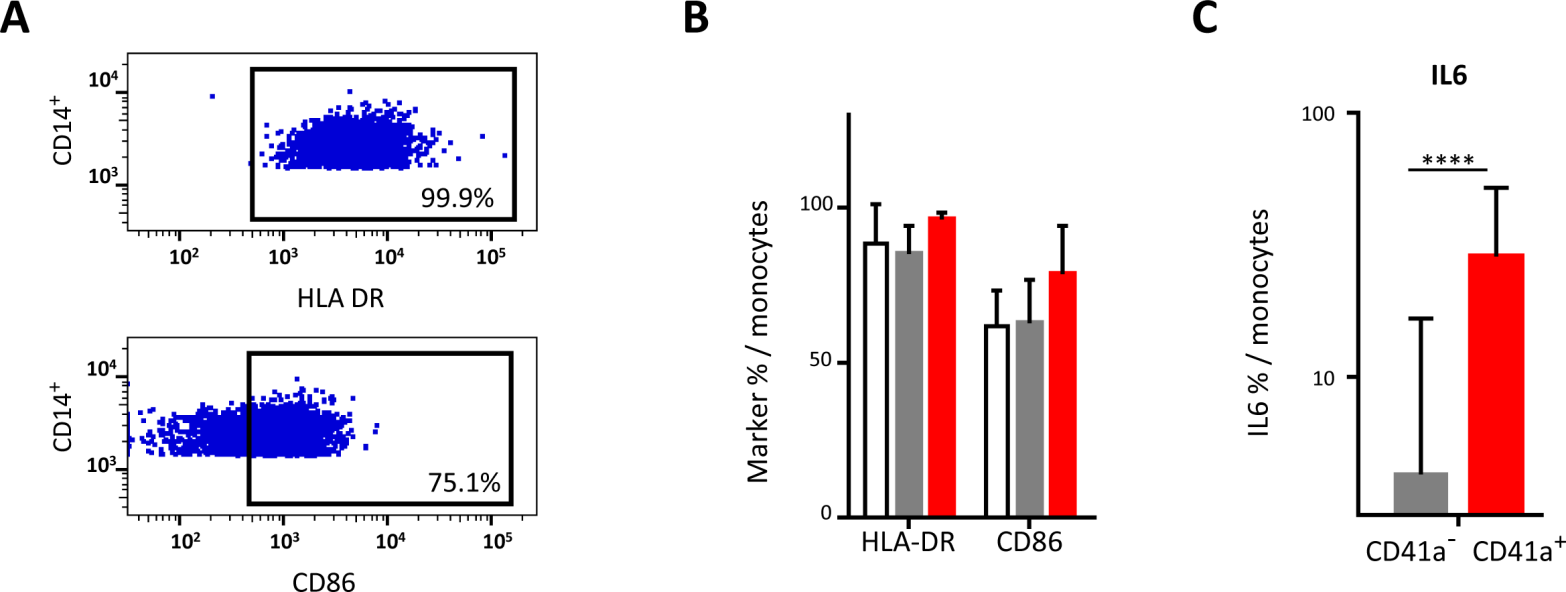
Impact of the binding of CD41a^+^ EVs to monocytes. Monocytes were cocultured for 18 hours with known numbers of CD41a^+^ EVs at a ratio of 1:20 (CD14^+^ cells: CD41a^+^ EVs). Cells from 17 HDs were used in seven experiments. **(A)** Example of the gating strategy. **(B)** The percentages (mean ± SD) of CD14^+^ cells expressing HLA-DR or CD86 were determined in the absence (white bars) or presence of EVs (CD14^+^CD41a^+^, red bars and CD14^+^CD41a^-^, gray bars). **(C)** IL6 secretion was assessed by intracellular staining of CD14^+^ cells cocultured for 18 h with EVs (CD14^+^CD41a^+^, red bars and CD14^+^CD41a^-^, gray bars). The *P* values shown were obtained in Wilcoxon’s tests. ****P<0.0001.

We studied the expression of CD40 and TLR9 on the surface of BLs following interaction between these cells and CD41a^+^ EVs (**Figure 3A**). BLs that interacted with CD41a^+^ EVs had significantly higher levels of CD40 expression than those that did not interact with EVs (54.87 ± 34.53% vs 30.46 ± 37.29% respectively, *P*<0.001) (**Figure 4B**). By contrast, TRL9 expression was unaffected (**Figure 3B**). All the B-cell subpopulations studied, including transitional B cells, naïve B cells, memory B cells, plasmablasts, and marginal zone B cells, interacted with CD41a^+^ EVs (**Figure 3C**).

The interaction of these EVs with plasma cells led to the production of polyclonal immunoglobulins. Immunoglobulin production levels after interaction with CD41a^+^ EVs were highest for IgM (124,233 ± 85,429 pg/ml), followed by IgG (112,607 ± 91,809 pg/ml for all IgG) (**Figure 3D**). Following interaction with CD41a^+^ EVs, the levels of IgA and IgE produced were lower than those of IgM and IgG (**Figure 4C**), IgA: 6,537 ± 2429 pg/ml; IgE: 18.6 ± 16.8 pg/ml.

### Interaction of CD41a^+^ EVs with monocytes

Monocytes were the immune cells with the highest rates of interaction with CD41a^+^ EVs, but this interaction did not affect the expression of the markers studied, such as HLA-DR and CD86 (**Figures 4A, B**). However, cells that interacted with CD41a^+^ EVs secreted more IL6 than those that did not interact with these EVs (42.92 ± 25.81% vs 4.27 ± 12.38%, *P*<0.001) (**Figure 4C**).

**Figure 5:**
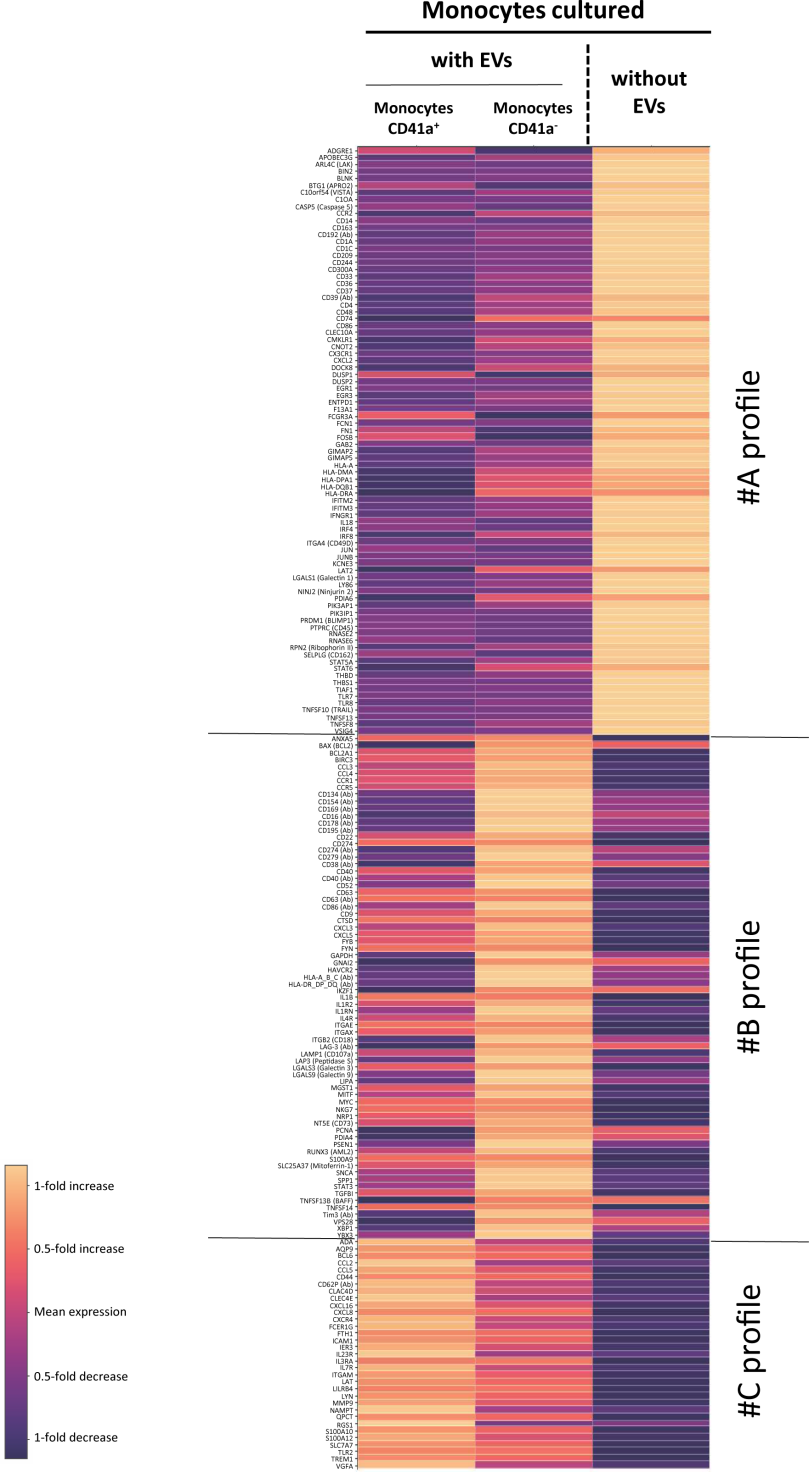
Comparison of RNA and protein levels between CD41a^+^ monocytes, CD41a^-^ monocytes and monocytes not incubated with EVs. RNAseq and protein analysis were performed in *n*=12 assays. Analyses were performed and heatmaps were generated with SeqGeq software. Heatmaps were established with the ViolinBox plug-in (version 5.1.11, www.flowjo.com/exchange/#/plugin/profile?id=13) developed by Luthy J., Taylor I., Spidlen J. This plug-in is based on Poggiali’s D algorithm. For each gene and protein, mean expression in the three sets of conditions (monocytes without EVs, monocytes that had interacted with CD41a^+^ EVs, and monocytes cultured with EVs but without interaction with CD41a^+^ EVs) was calculated with ViolinBox. Only genes and proteins with a *Q*<0.05 (Kruskal-Wallis test) were considered to be differentially expressed. The colors used indicate the deviation from this mean. Light hues indicate an increase in expression relative to the mean of the three conditions, whereas dark hues represent a decrease in expression relative to the mean of the three conditions. Expression levels cannot be compared between different genes or proteins in this representation.

For assessment of the reprogramming of monocytes by CD41a^+^ EVs derived from PCs, we used an oligonucleotide-conjugated anti-CD41a antibody to identify CD41a^+^ EVs before culture (**Supplementary Figure 1**). We allowed these EVs to interact with purified monocytes for 18 h. We then determined the levels of 20 proteins and assessed the expression of 399 genes in monocytes that did and did not bind to CD41a^+^ EVs (left column, **Figure 5**).

In this assay, we were able to detect an effect of all interacting EVs and, more particularly, CD41a^+^ EVs. We identified three groups of genes and proteins displaying differential expression between cells cultured with and without these EVs. All EVs induced a decrease in the expression of several genes encoding immunoregulatory molecules, such as *CD209*, *CD244*, *CD300a*, *CD37*, *CD48*, *CD74* and *CD86* (**Figure 5**, profile A). By contrast, CD41a^+^ EVs appeared to have significantly less regulatory activity than other EVs (**Figure 5**, profile B). However, the interaction of CD41a^+^ EVs with monocytes appears to result in a specific activation of several other genes: *CCL2*, *CCL5*, *CXCL16*, *CXCL8* and *CXCR4*. (**Figure 5**, group C).

## Discussion

Significant interactions are known to occur between EVs and monocytes (25, 46). These interactions depend on the numbers of EVs present, their cellular origin and the molecules present on their surface, as these molecules direct the interactions (22, 39).

The numbers of EVs present in transfusion products vary considerably and depend, above all, on the physiological state of the donor (22, 29, 50–55), but the method used to prepare the platelet concentrates may also have an effect (56–58). Our findings indicate that the number of EVs has a crucial effect in determining their immunological effect on the immune system, as it governs the type of effectors and antigen-presenting cells affected. One of the key limitations of this study model is that it does not take platelet polytransfusion into account.

It is very difficult to estimate the ratio of EVs to cells for use in functional tests to ensure that conditions are as close as possible to real-life conditions and the impact of CD41a^+^ EVs delivered during transfusion. The concentrates used in transfusions are obtained by apheresis or from multiple donors, and the number of units transfused depends on the severity of the patient’s condition and body mass. Blood cell counts in each patient should also be taken into account. We selected the ratio for functional tests based on data in our possession concerning the number of CD41^+^ EVs present in platelet concentrates and the number of transfusion units administered to polytransfused patients with hematological malignancies (12, 29). For this study, we used a 1:20 ratio (cells: EVs). This ratio was appropriate regardless of the elements considered, for all cell types studied. We estimate the mean number of CD41a^+^ EVs per transfusion at between 5.32x10^9^ and 3.3x10^10^ CD41a^+^ EVs. The maximum numbers recorded were between 9.5x10^9^ and 5.9x10^10^ CD41a^+^ EVs.

Platelets and their EVs express a restricted number of ligand-receptor pairs, potentially accounting for the increase in interaction rates with the number of EVs observed for monocytes and BLs. In this study, we were not able to assess the full range of mechanisms of interaction between different cell types, particularly for interactions with phagocytic cells. We can assume that phagocytosis is one of the mechanisms of interaction, as we observed identical levels of interaction between CD41a^+^ EVs and CD39^+^ EVs for monocytes (59). Nevertheless, we were able to observe differences in the interaction of EVs with these cells depending on the cellular origin of the EVs (data not shown). This difference suggests several mechanisms of interaction for phagocytic cells, including a specific interaction associated with ligand/receptor pairs.

CD40/CD154 molecules are poorly expressed on CD41a^+^ EVs (**Supplementary Figure 2**); we therefore studied the OX40/OX40L axis with a blocking assay, as previously described (39, 59). Our results suggest that OX40 and OX40L are not involved in these interactions (data not shown). CD41a^+^ EVs also express other markers and ligand/receptor pairs that may be involved in these interactions (**Supplementary Figure 2**).

Following the interaction of CD41a^+^ EVs with CD4^+^ TLs, we observed an increase in the expression of PD1, ICOS, CCR6 and CD25 on the surface of TLs. Unlike PDL1, PD1 has not been reported to be expressed by platelets (60). The higher levels of PD1 expression on CD4^+^ TLs may, therefore, be linked to interactions with CD41a^+^ PDL1^+^ EVs. Interactions with CD41a^+^ EVs may underline this activation, as reported for platelets (61). The functional effect of CD41a^+^ EVs was investigated for CD4^+^ TLs only. The percent interaction was identical in the various TL subpopulations but given the much lower frequency of CD8^+^ TLs than of CD4^+^ TLs, it was not possible to investigate function effects on CD8^+^ TLs due to the small volume of blood (7 ml) collected from healthy donors at blood donation centers. This imposed sampling volume made it impossible to present reliable functional results for the TLs interacting with CD41a^+^ EVs (**Supplementary Figure 3**).

In addition to protein reprogramming via the transfer of molecules present on EVs, changes in gene expression were observed that may result from miRNA transfer, as previously described (34, 36, 62–65), with microRNA-34a-5p already shown to be directly linked to an increase in PD1 expression (65). This interaction led to functional activation, with an increase in the production of IL2, TNF and IL17, consistent with a hypothesis of general activation linked to CD41a^+^ EVs, as reported for platelets (61).

As for BLs, we found that this activation (**Figure 3**) was probably strongly linked to CD41a^+^CD40^+^ EVs (**Supplementary Figure 2**). We previously showed that EVs appeared to have a polyclonal stimulatory effect, with antibody production following interaction (22, 59). We have also described this effect on humoral immunity in a model in which mice were transfused with heterologous EVs *in vivo* (22). This activation affected all immunoglobulin isotypes and all subpopulations of BLs (**Figure 3C**). The exact mechanism involved remains unknown, but the heterogeneity of EVs may account for these unexpected results. The CD40L present on CD41a^+^ EVs may interact directly with BLs (**Supplementary Figure 2**) (22, 66). EVs also contain CpG dinucleotides (mitochondrial CpG), which can be detected by TLR9 on BLs (**Figure 3A**) (17).

Monocytes displayed significantly higher rates of interaction with CD41a^+^ EVs than other cells and have been implicated in leukemia progression (44, 45). We therefore performed a more detailed investigation of the impact on monocyte gene expression of the interaction of these cells with EVs, and with CD41a^+^ EVs in particular. To this end, we developed a new method for studying the interactions of EVs with cells. This original multi-omics study was performed after incubating EV-labeling oligonucleotide-conjugated antibodies with purified monocytes for 18 h. No public database findings for platelet transfusion effects were available for comparison with our results, but these results provided considerable insight into the cellular reprogramming of monocytes after interaction with EVs or CD41a^+^ EVs.

The most important modulation observed after interaction with EVs was a decrease in *CD86* expression. The downregulation of this gene encoding a costimulatory molecule indicates that these EVs may have immunoregulatory effects on monocytes. A decrease in *DC-SIGN* (*CD209*) expression was also observed. The DC-SIGN on macrophages recognizes high-mannose type N glycans, binding to the members of this class of PAMPs (pathogen-associated molecular patterns) with high affinity; it may also decrease phagocytosis (66).

We also observed a decrease in the expression of *2B4* (*CD244*) following interactions. This molecule has been implicated in the immunosuppressive phenotype of monocytes (67). It has also recently been shown that the targeted deletion of CD244 on monocytes promotes the differentiation of these cells into antitumorigenic macrophages (68). This depletion would also improve antitumor responses in patients on anti-PDL1 antibody-based immunotherapy (69). The major role of CD244 was supported by the downregulation of another member of the CD2 subfamily: *CD48*. We also noted a decrease in the expression of other markers involved in immunoregulation, such as CD37. CD37 is already targeted in antitumor immunotherapy and its absence from lymphomas is associated with a poor prognosis (69, 70). This was one of the most interesting results of our analysis, as we know that CD37 is downregulated on the activation of monocyte-derived DCs and that CD37^lo^ DCs have a greater capacity for the activation of naive TLs (71). We also observed a decrease in the expression of *CD300a*. This downregulation is of particular interest in leukemia patients undergoing polytransfusion, as high levels of CD300a have been associated with a poor prognosis in this context (72). *CD74* expression was also downregulated after the interaction of EVs with monocytes, confirming the impact of these EVs on monocytes and indicating a possible decrease in the ability of monocytes to migrate after interaction (73). Finally, we also observed a decrease in the expression of *CXCL2* that might play a key role in the escape of acute myeloid leukemia from treatment (74).

Interactions with CD41a^+^ EVs led to monocyte activation, with higher levels of expression for chemokines and receptors, particularly *CXCL8* (**Figure 5**, group C). We observed no increase in the level of IL8 secretion, which was already high in the basal state. The most striking feature confirming this reprogramming by platelet EVs was the change in the levels of the CD62P protein. However, this activation was much less marked than that observed following interactions with total EVs (**Figure 5**, group B), for which we observed the transfer of large amounts of the CD134, CD154, CD169, CD16, CD178, CD195, CD274, CD279, CD38, CD40, CD63, CD86, LAG-3 and HLA Class I or II proteins, and the induction of expression of many genes potentially associated with cell activation and chemoattraction.

At this stage in our exploration of the interaction of monocytes with CD41a^+^ EVs and given the questions posed, we decided to limit the use of AbSeq antibodies to immunoregulatory molecules only. This resulted in a voluntary lack of data for certain monocyte subpopulations. However, multi-omics data suggested the involvement of several different groups of monocytes (**Supplementary Figures 4**–**7**).

In addition to the transfer of molecules present on EVs, changes in gene expression may be linked to miRNA transfer (34, 36, 59–61). Several miRNAs, including miR-126, have already been implicated in such transfers (36, 59). miR-126 targets the VEGF pathway during its transfer from endothelial EVs to monocytes (50). The transfer of this miRNA is of potential interest because an increase in *VEGFA* expression was observed during interactions with CD41a^+^ EVs.

These results support the hypothesis that extracellular vesicles — and particularly EVs expressing CD41a — present in platelet concentrates can interact with immune system cells, rapidly modulating the phenotype and function of these cells.

One of the limitations of this pioneering study is that it does not cover all the cells of the immune system. The complexity of the EV phenotype and the number of possible cell targets naturally make it impossible to explore every eventuality. Nevertheless, as we show here, the use of multi-omics systems opens up new possibilities for rapidly exploring these interactions.

Platelet transfusion is a major component of the management of hematological malignancies. However, transfusions are not immunologically innocuous. These data are important for vigilance regarding the immunological effects of transfusion on hemopathies and the immunotherapies used to treat them.

## Data Availability

The original contributions presented in the study are included in the article/**Supplementary Material**. Further inquiries can be directed to the corresponding author.
