# Supplementary material for: Interactions with and activation of immune cells by CD41a+ extracellular vesicles
Source: Front Immunol. 2025 Feb 14;16:1509078. doi: 10.3389/fimmu.2025.1509078 (PMC11868057; doi:10.3389/fimmu.2025.1509078)
Supplement: Supplementary file 1 [file DataSheet1.docx]

**Interactions with and activation of immune cells by CD41a^+^ microparticles**

Marie Tamagne, Mehdi Khelfa, Souganya Many, Deborah Neyrinck-Leglantier, Adèle Silane Delorme, Marion Klea Pinheiro, Muriel Andrieu, Sabine Cleophax, France Pirenne and Benoît Vingert

**Supporting information**

**Supplemental Table 1: Antibody panel for flow cytometry**, page 2

**Supplemental Table 2: AbSeq-antibody panel for the single-cell multi-omics assay**, page 3

**Supplemental Figure 1: Sample preparation for the multi-omics assay**, page 4

**Supplemental Figure 2: Expression of markers and ligand/receptor pairs on EVs expressing CD41a**, page 5

**Supplemental Figure 3: Examples of cytokine production by CD4^+^ T lymphocytes (TLs) after interaction with CD41a^+^ EVs**, page 6

**Supplemental Figure 4: Clustering of monocyte (CD14^+^ cells) mRNA and protein levels between cells cultured with and without CD41a^+^ EVs**, page 7

**Supplemental Figure 5: Comparison of RNA and protein levels between different phenotypic clusters of monocytes used for CD41a^+^ EV interaction assays**, page 8

**Supplemental Figure 6: Comparison of RNA and protein levels between different phenotypic clusters of monocytes that had interacted with CD41a^+^ EVs**, page 9

**Supplemental Figure 7: Comparison of RNA and protein levels between different phenotypic clusters of monocytes cultured with CD41a^+^ EVs but without interaction with these EVs**, page 10

**Supplemental Table 1:** Antibody panel for flow cytometry

**Supplemental Table 2:** AbSeq**-**antibody panel for the single-cell multi-omics assay

**Supplemental Figure 1: Sample preparation for the multi-omics assay.** (A) EVs isolated from HD platelet concentrates were stained by incubation with oligonucleotide-conjugated anti-CD41a antibody for 60 min at 4°C. (B) Sorted monocytes were cultured without EVs (I) or with EVs from 4 different HD platelet concentrates (II, III, IV and IV) for 18 h at 37°C. (C) For each set of conditions, staining was performed by incubation with a sample tag and with 20 oligonucleotide-conjugated membrane protein antibodies for 1 h at 4°C. (D) All sets of conditions were then pooled for loading on a Rhapsody cartridge.


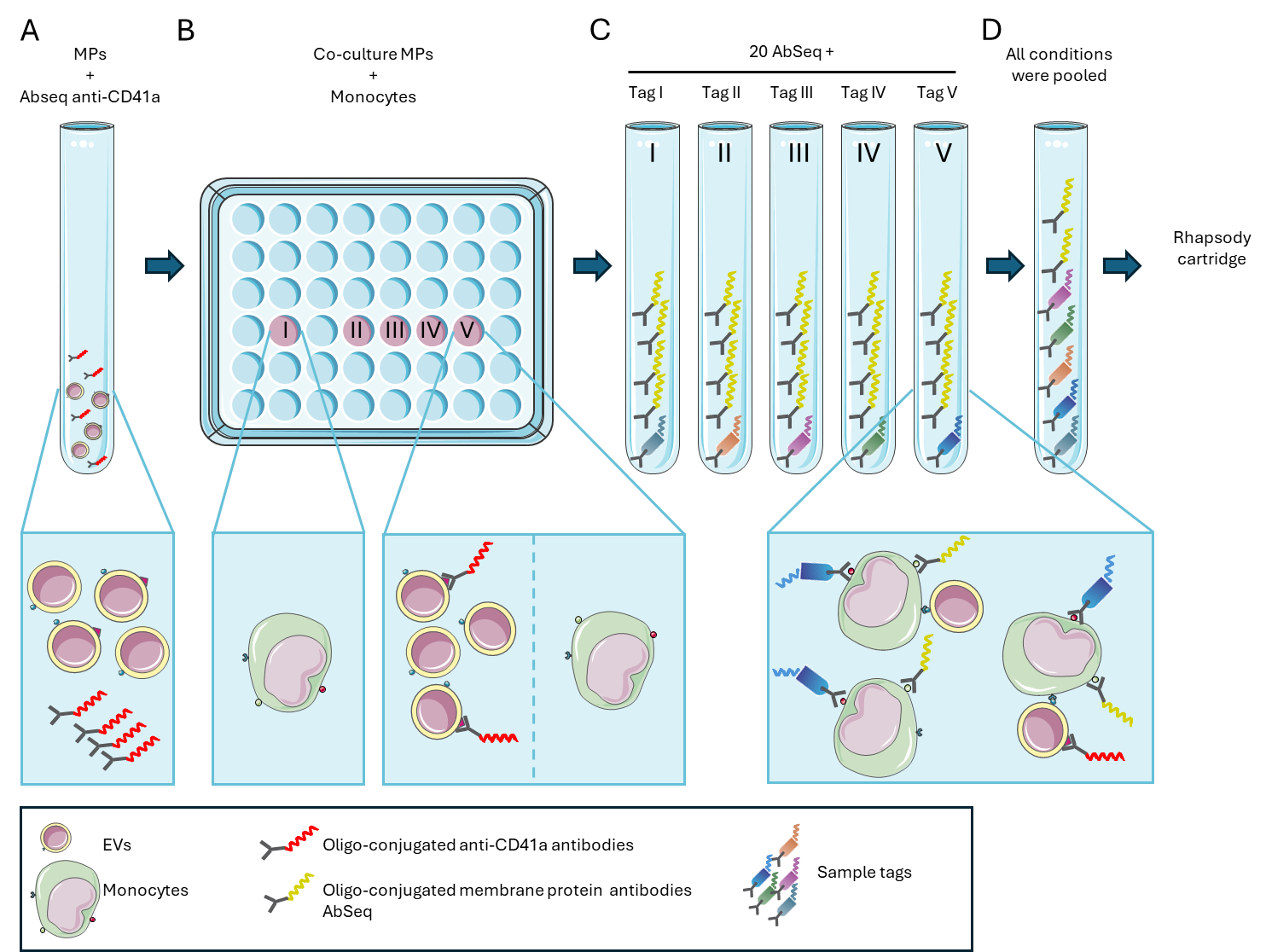

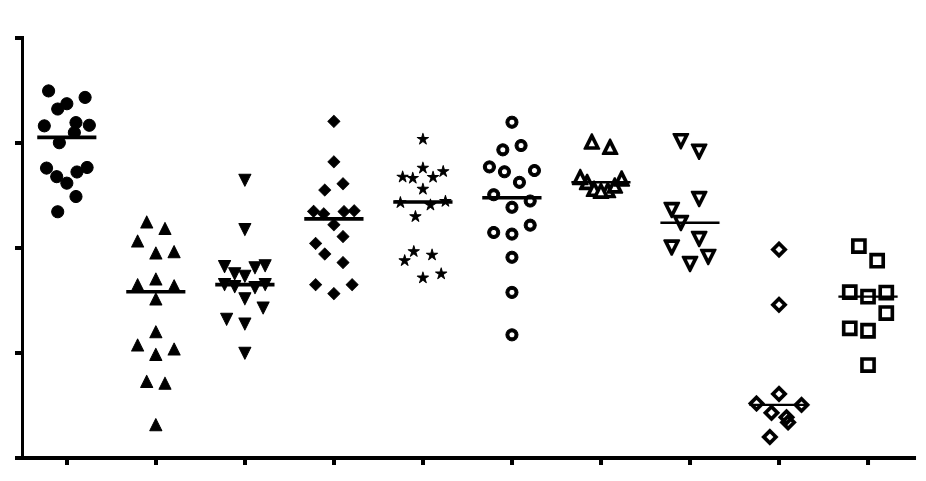


CLEC2

OX40L

CD107a

CD80

CD86

CD73

CD62

OX40

CD40L

CD40

100

10

1

0.1

0.01

% EVs / CD41a^+^ EVs

**Supplemental Figure 2: Expression of markers on EVs expressing CD41a.** The percentages of CD41a^+^ EVs expressing CD62P, CD73, CD80, CD86, CD107a, or CLEC2 were determined by flow cytometry. Marker expression was assessed for EVs from 16 HDs in six experiments. The percentages of CD41a^+^ EVs expressing OX40L, OX40, CD40L or CD40 were determined by flow cytometry. Marker expression was assessed for EVs from 9 HDs in three experiments.

**
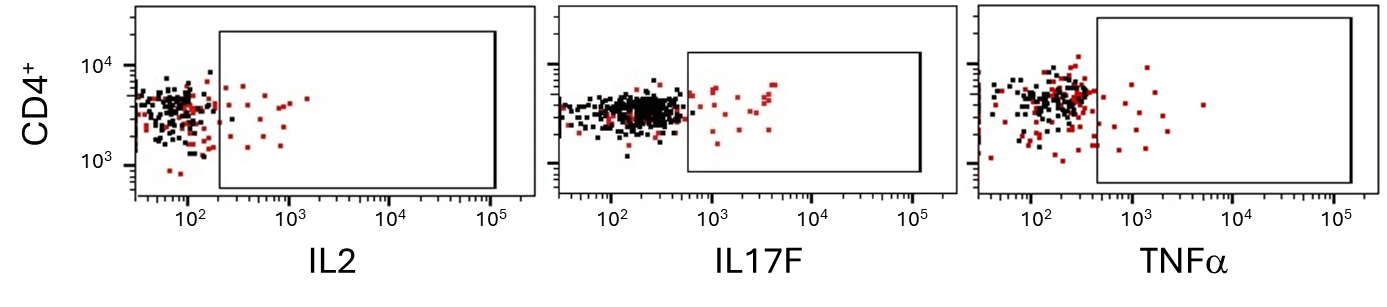
**

**Supplemental Figure 3: Examples of cytokine production by CD4^+^ T lymphocytes (TLs) after interaction with CD41a^+^ EVs.** Overlay image of the cytokine production of CD4^+^ TLs without culture with CD41a^+^ EVs (black dots) and that ofCD4^+^ TLs cultured with CD41a^+^ EVs (red dots). Cytokine production by CD4^+^ TLs cultured with CD41a^+^ EVs was assessed with CD3^+^CD4^+^CD45RA^-^CD41a^+^ EVs.

**Supplemental Figure 4: Clustering of monocyte (CD14^+^ cells) mRNA and protein levels between cells cultured with and without CD41a^+^ EVs**. Single-cell RNAseq and protein analysis were performed for *n*=12 CD41a^+^ EV interaction assays. tSNE visualization of cells, each color representing a different phenotypic cluster. Analyses were performed with SeqGeq software (v1.8, BD Biosciences) and differentially expressed genes and proteins were identified in Kruskal-Wallis tests. Only genes and proteins with a *Q*<0.05 were considered to be differentially expressed. The classic Phenograph plug-in (version 4.0.5) was used to establish clusters (www.flowjo.com/exchange/#/plugin/profile?id=1). This plug-in was developed by Spidlen J., Taylor I., Luthy J., and Hinson D and is based on the algorithm established by Levine J. and Simonds E.F.


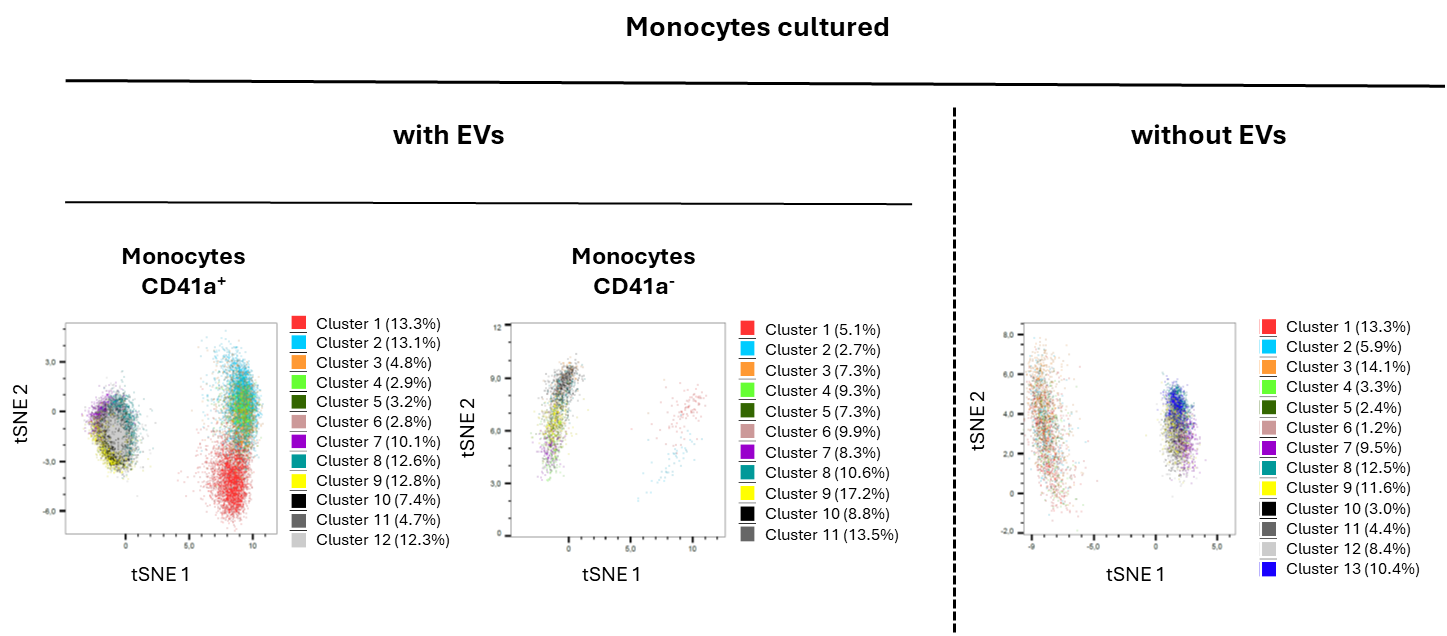


**
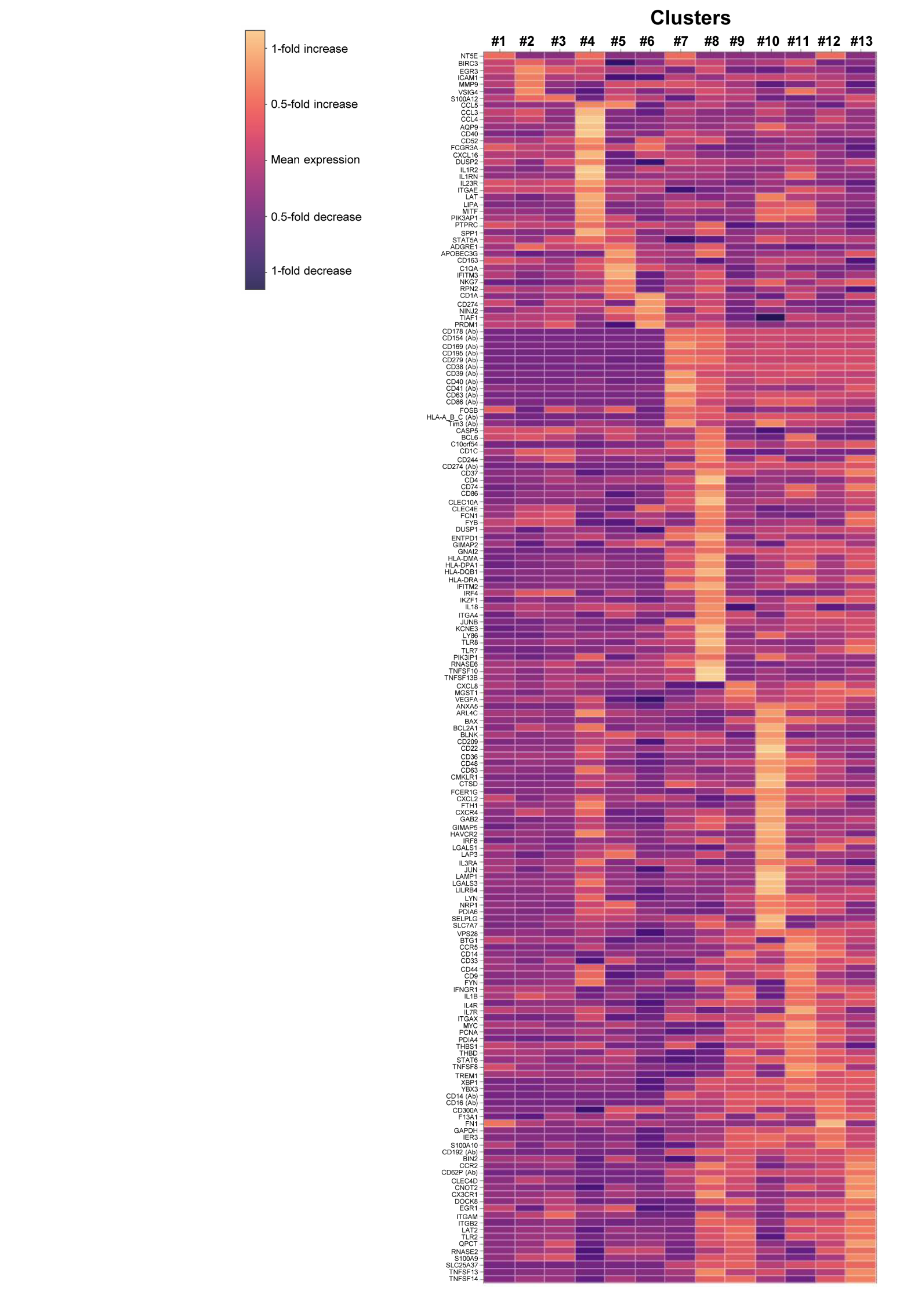

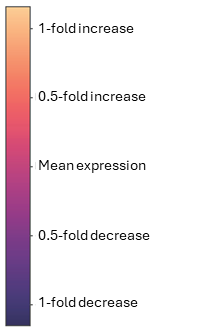
**

**Supplemental Figure 5: Comparison of RNA and protein levels between different phenotypic clusters of monocytes used for CD41a+ EV interaction assays.** Monocytes were not incubated with CD41a+ EVs. RNAseq and protein analysis were performed in n=12 assays. Analyses were performed and heatmaps were generated with SeqGeq software. Heatmaps were established with the classic ViolinBox plug-in (version 5.1.11, www.flowjo.com/exchange/#/plugin/profile?id=13) developed by Luthy J., Taylor I., Spidlen J. This plug-in is based on Poggiali’s D algorithm. For each gene and protein, the mean expression of the 13 clusters was calculated with ViolinBox. Only genes and proteins with a Q<0.05 (Kruskal-Wallis tests) were considered to be differentially expressed. The colors used indicate the deviation from this mean. Light hues represent an increase in expression relative to the mean of the 13 clusters, whereas dark hues represent a decrease in expression relative to the mean of the 13 clusters.

**
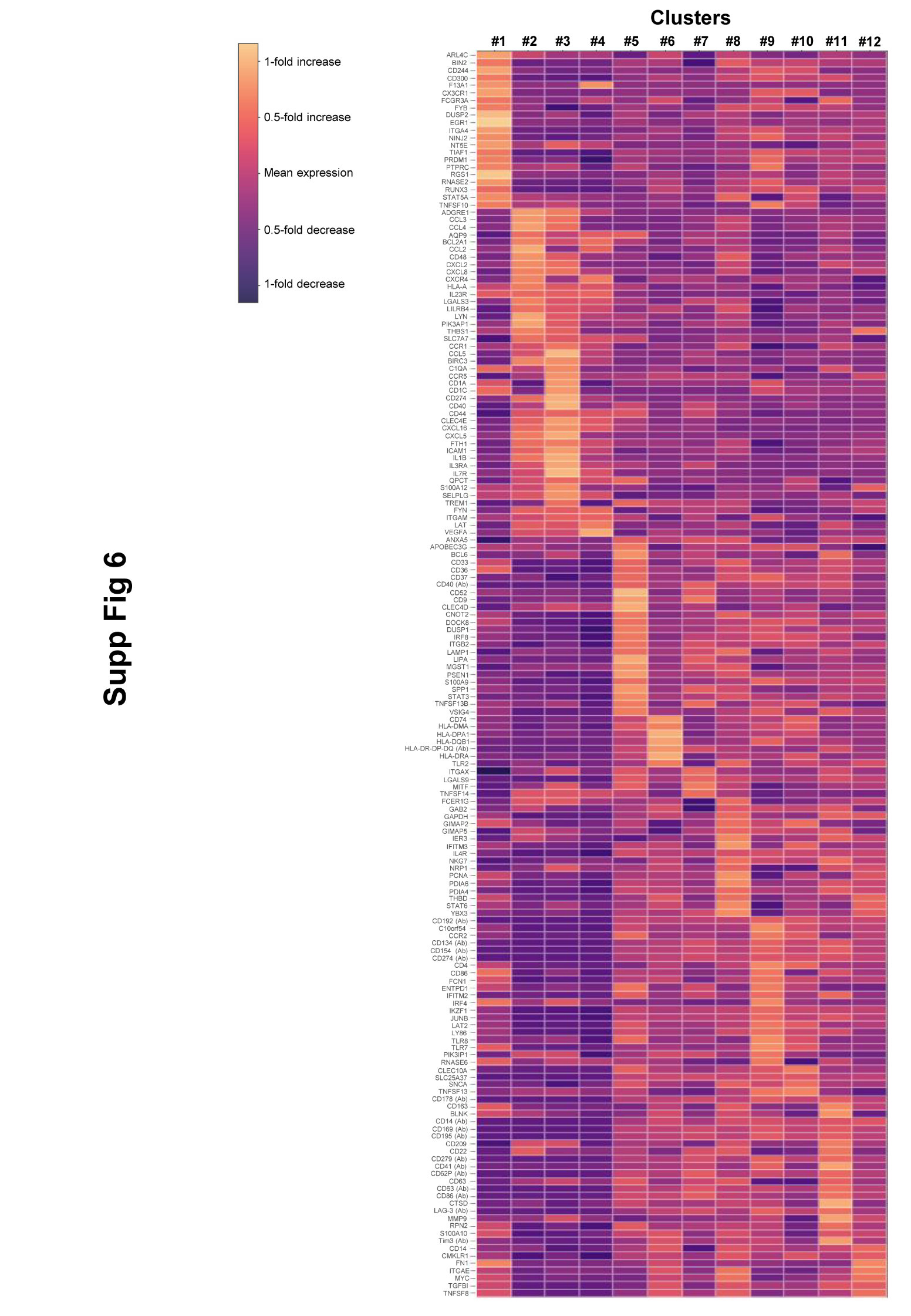

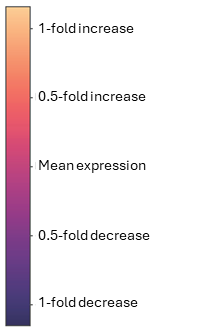
**

**Supplemental Figure 6: Comparison of RNA and protein levels between different phenotypic clusters of monocytes interacting with CD41a+ EVs.** RNAseq and protein analysis were performed in n=12 assays. Analyses were performed and heatmaps were generated with SeqGeq software. Heatmaps were established with the classic ViolinBox plug-in (version 5.1.11, www.flowjo.com/exchange/#/plugin/profile?id=13) developed by Luthy J., Taylor I., Spidlen J. and based on Poggiali’s D algorithm. For each gene and protein, mean expression in the 12 clusters was calculated with ViolinBox. Only genes and proteins with a Q<0.05 (Kruskal-Wallis tests) were considered to be differentially expressed. The colors indicate the deviation from this mean. Light hues indicate an increase in expression relative to the mean of the 12 clusters whereas dark hues indicate a decrease in expression relative to the mean of the 12 clusters.


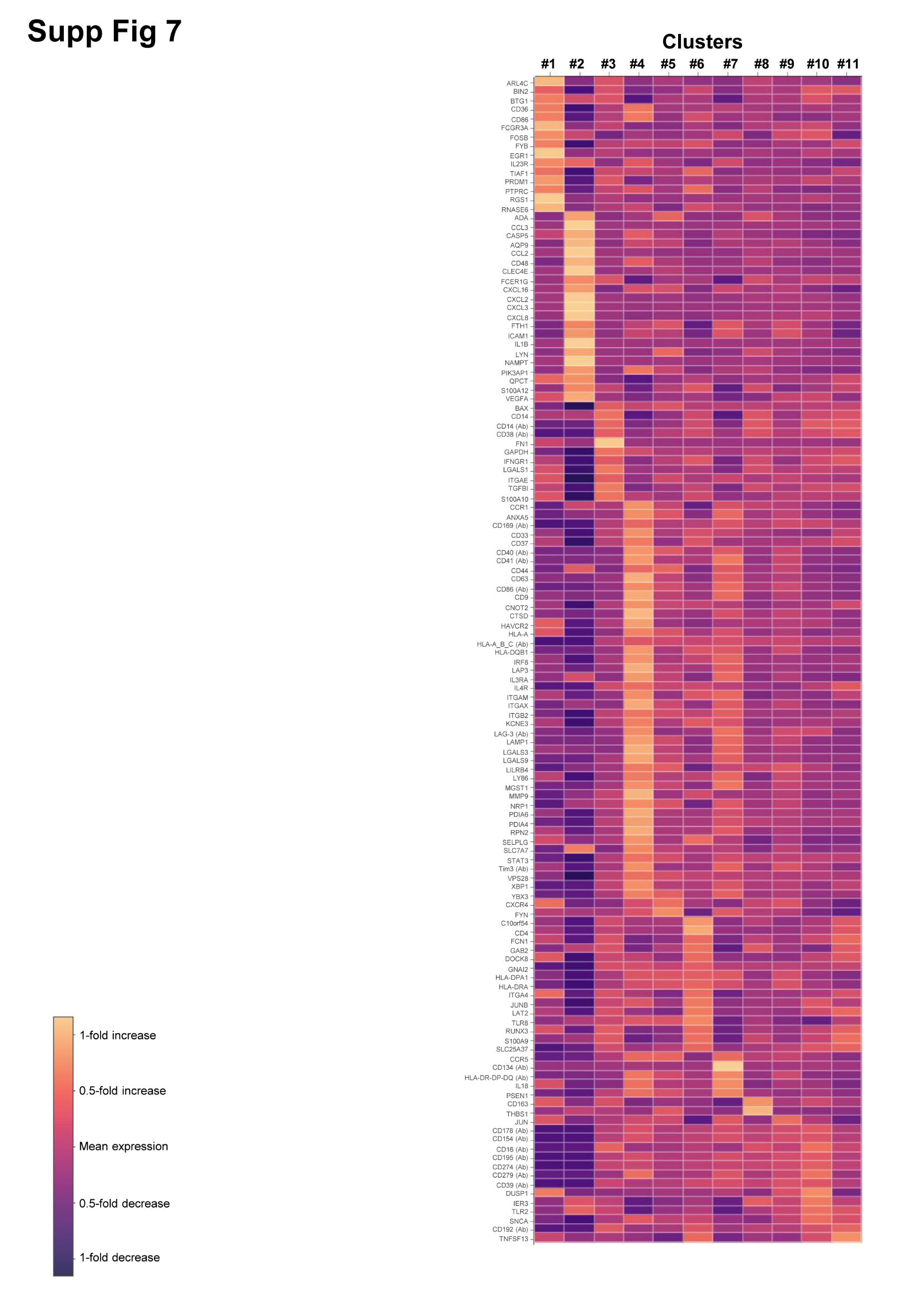
**
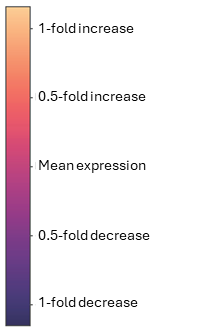
**

**Supplemental Figure 7: Comparison of RNA and protein levels between different phenotypic clusters of monocytes cultured with CD41a+ EVs but without interaction with these EVs.** RNAseq and protein analysis were performed in n=12 assays. Analyses were performed and heatmaps were generated with SeqGeq software. Heatmaps were established with the classic ViolinBox plug-in (version 5.1.11, www.flowjo.com/exchange/#/plugin/profile?id=13) developed by Luthy J., Taylor I., Spidlen J. and based on Poggiali’s D algorithm. For each gene and protein, mean expression in the 11 clusters was calculated with ViolinBox. Only genes and proteins with a Q<0.05 (Kruskal-Wallis tests) were considered to be differentially expressed. The colors indicate the deviation from this mean. Light hues indicate an increase in expression relative to the mean of the 11 clusters whereas dark hues represent a decrease in expression relative to the mean of the 11 clusters.
